# Supplementary material for: Are carbon emissions trading and green financial instruments synergistic? -Comprehensive quantitative research based on content analysis
Source: PLoS One. 2024 Mar 7;19(3):e0298601. doi: 10.1371/journal.pone.0298601 (PMC10919601; doi:10.1371/journal.pone.0298601)
Supplement: S1 Data — (ZIP) [file pone.0298601.s001.zip › Data/地级市Z_F工作报告.zip!/╕ú╜¿/─■╡┬╩╨/└┤╫╘╣1⁄2╓┌║┼í╛┬φ┐╦╩2╛▌═°í┐_2012.docx]

各位代表：

现在，我代表宁德市人民政府向大会作政府工作报告，请予审议，并请各位政协委员和列席人员提出意见。

一、2011年及过去五年政府工作回顾

2011年，在省委、省政府和市委的正确领导下，我市各级政府深入贯彻落实科学发展观，全面实施环三发展战略，以“五大战役”和“五个年”活动为抓手，全力推动宁德科学发展、跨越发展，顺利完成市二届人大六次会议确定的主要目标任务，实现“十二五”良好开局。全市生产总值933.71亿元、增长15.2%；财政总收入83.42亿元、增长32.8%，地方级财政收入54.3亿元、增长34.1%；城镇居民人均可支配收入19314元、增长14.9%，农民人均纯收入7756元、增长18.5%；物价涨幅低于全省平均水平。

（一）抓特色、调结构，产业发展提速增效

现代农业稳步发展。农林牧渔业总产值290亿元、增长5%。粮播面积和粮食产量保持稳定。农业产业不断发展壮大，新增1家国家级、13家省级农业产业化重点龙头企业。完成造林面积124.6万亩，相当于过去六年造林总数。完成土地整理3.7万亩，加固病险水库54座，除险加固海堤21.5公里。防灾减灾工作扎实有效，农机、气象、水文等服务保障更加有力。

工业经济快速增长。工业总产值1509亿元、增加值372亿元，分别增长26.6%和25%。新增规模以上企业49家，达1083家，其中产值亿元以上企业新增68家，达312家。规模以上工业总产值1386.77亿元、增长28.4%，增加值361.73亿元、增长26.9%，连续七年居全省首位。

现代服务业加快发展。第三产业增加值320亿元、增长9.5%。新增销售额过5000万元的商贸流通企业5家，一批国内外大型流通企业进驻我市。实施“万村千乡市场工程”，加快农贸市场改造，建成市级副食品基地51个，全市社会消费品零售总额273.94亿元、增长17.3%。成功举办首届宁德世界地质公园文化旅游节，加强旅游推介和服务，全年接待游客852万人次，旅游收入70亿元，分别增长25%和38%。

（二）抓项目、打基础，发展后劲持续增强

“五大战役”成效突出。全市上下掀起大干“十二五”开局之年的热潮，全年完成投资476亿元，占年度计划的120%。其中，完成重点项目建设战役投资252亿元、新增长区域发展战役投资208亿元、城市建设战役投资87亿元、小城镇改革发展战役投资70亿元、民生工程战役投资41亿元，分别占年度计划的110%、122%、138%、140%和104%。

“三维”对接亮点纷呈。举办10场招商活动，累计签约“三维”项目351项、总投资1542亿元，其中合同项目297项、总投资1290亿元，已动工161项，动工率54.2%。开展“重点项目攻坚年”活动，一批重大“三维”项目加快推进，福建鞍钢（宁德）钢铁基地列入国家《钢铁工业“十二五”发展规划》，宁德（漳湾）临港工业区获省政府批准成立，宁德核电站、中海油海西宁德工业区加快建设，福建益联远大可持续建筑生产线等一批生产性项目动工建设。

基础设施更趋完善。开展“交通建设会战年”活动，宁武高速公路、合福铁路、白马港铁路支线和一批码头泊位工程加快建设，京台高速公路、沈海复线宁连段和漳湾8#、9#泊位开工建设，国省干线改扩建完成184公里，宁古二级公路提前一年通车。能源保障不断强化，新增110千伏输变电线路84.2公里，宁德核电站至宁德变电站500千伏输电线路工程全线贯通。信息化步伐加大，“智慧宁德”和“无线城市”建设加快推进，新增接入网光缆5.6万芯公里，行政村光缆通达率达95%。

改革开放步伐加快。投融资体制不断创新，代建、代管、BT、BOT等模式得到积极应用。企业上市取得新进展，31家企业被确定为省上市后备企业，6家企业进入辅导期。林权配套改革深入推进，林权发证率87.3%，入户率73.3%。外资项目成倍增长，新批外资项目30项，合同外资额2.7亿美元、增长2.1倍，验资口径实际利用外资9512万美元、增长34%。外贸出口强势增长，出口总额14亿美元、增长43.7%。宁台合作不断深化，成功举办第二届海峡两岸电机电器博览会和“海峡论坛·陈靖姑文化节”，宁德新能源锂离子电池一期竣工投产，我市最大的台商投资项目——台湾义联集团联德60万吨镍合金项目开工建设。

环境保护力度加大。节能减排责任制和环保监管“一岗双责”有效落实，主要污染物排放得到控制。市生活垃圾焚烧发电厂、北区污水处理厂建成投产，各县（市、区）污水处理厂稳定运行，污水处理率达82.7%。重点流域水环境综合整治深入推进，县级以上集中式生活饮用水源和主要流域水质功能达标率达99%。生态示范创建活动深入开展，屏南入选首批“国家级绿色能源示范县”，全市生态环境质量继续保持优良水平。

（三）抓规划、重统筹，城乡建设协调推进

中心城市功能持续提升。开展“城市功能提升年”活动，完成宁德城市总体规划修编，编制和实施各类“十二五”专项规划。完成中心市区蕉城南北路、福宁南路、闽东路、万安路沥青路面改造，同步完成“平改坡”、人行道改造、管线下地和户外广告牌整治。宁德万达广场等城市综合体加快建设。积极创建国家级园林城市，城市建成区绿化覆盖率达40.1%，人均公共绿地面积达13.6平方米。“清违”行动取得阶段性成效，拆除“两违”建筑面积4.42万平方米，清理非法围填海面积1050亩。市城市管理行政执法局正式成立，城市管理水平有效提升。

县域经济实力不断壮大。实施一系列扶持和促进县域经济发展的政策措施，县域经济发展质效大幅跃升，县（市）地区生产总值、工业总产值、地方级财政收入占全市的比重分别达84%、83%和68%，福安、福鼎荣膺全省县域经济发展十佳县（市）。

小城镇改革发展加快推进。4个省级、15个市级小城镇综合改革建设试点力度加大，各级试点镇全面完成总体规划和一批控制性详规、专项规划编制工作。省级试点镇启动垃圾、污水处理等基础设施建设及“三旧”改造工程。

（四）抓民生、办实事，社会大局和谐稳定

惠民措施有效落实。开展“民生工程推进年”活动，努力提高人民群众幸福指数。全年发放城乡低保金1.9亿元，城乡医疗救助12.6万人次。新型农村社会养老保险全面实施，城镇居民社会养老保险开展试点。新建城市社区综合服务站11个、农村敬老院16所、居家养老服务中心（站）80个。保障性安居工程全面推进，完成投资15.5亿元，占年度计划的103.1%，建成和基本建成保障房6765套。农村基础设施不断完善，新建农村公路510公里，完成安保工程2700公里，建制村通客车率达87%。解决22.7万农村人口饮水安全问题。“造福工程”和地灾隐患点搬迁4584户2.02万人，全面兑现5年搬迁12.5万人的承诺。建成自然灾害避灾点2600个。就业再就业工作扎实开展，城镇登记失业率2.39%。加强主副食品价格调控，物价涨幅明显回落。出生人口政策符合率大幅提高，免费孕前优生健康检查全面推广，打击“两非”工作持续开展，全市人口自然增长率7.35‰。

社会事业持续进步。实施国家、省级科技计划项目41个，新认定高新技术企业13家。新改扩建22所公办幼儿园，新增4所省级达标高中，完成116所义务教育标准化学校建设。宁德职业技术学院通过教育部高职院校人才培养工作评审，闽东高级技工学校通过技师学院省级验收。宁德市医院晋升三级甲等医院。参加新型农村合作医疗农民243.1万人，平均参合率99.2%。扶贫政策有效落实，老少边岛地区和库区发展条件不断改善。市妇女儿童活动中心迁建工程扎实推进。市慈善总会正式成立，市社会福利中心一期土建封顶。民族和宗教工作进一步加强。人事、编制、统计、档案、外事、侨务、老龄、老干部、地方志、残疾人、关心下一代等工作取得新成绩。

社会管理切实加强。积极创新社会管理，严格落实社会稳定风险评估机制，全面贯彻信访“路线图”，积极构建“大调解”工作体系，大力推广福鼎市社会矛盾排查调处中心、福安市阳春社区“六贴近、六服务”机制、古田县平湖镇“三点一团”机制，多元化化解矛盾纠纷能力不断提升。“平安宁德”建设深入推进，社会治安综合治理和公共安全监管力度不断加大。人民群众对社会治安满意率达93.9%，对平安工作的党政重视认同率和知晓率分别达86.1%、77.4%，由2010年排名全省第八、第九位双双跃升至第五位。安全生产“一岗双责”全面落实，全市安全生产形势保持稳定。应急管理体系逐步健全，应对突发公共事件能力逐步提升。“五五”普法通过省级验收，“六五”普法启动实施。双拥支前工作取得新成效。人防、海防、反走私、国防动员、民兵预备役等工作取得新成绩。

（五）抓创建、促提升，文化事业加快发展

精神文明建设切实加强，群众性精神文明创建活动深入开展，公民道德水平和文明程度进一步提升。隆重举办纪念建党90周年、辛亥革命100周年系列文化活动。公共文化服务体系不断完善，启动市图书馆建设，新建乡镇综合文化站22个，新建、改造农家书屋458个，农村有线广播“村村响”工程、有线数字电视整体转换全面推进。文化遗产保护力度加大，一批非物质文化遗产列入国家和省名录。体育事业稳步发展，全民健身运动深入开展，市老年人体育中心开工建设，新建农民体育健身工程452个、青少年校外体育活动中心10个。

（六）抓服务、提效能，政府建设不断加强

全面推进依法行政，切实加强效能建设。依法贯彻执行市人大及其常委会的决定决议，认真办理人大代表的建议意见和人大常委会的审议意见，全力支持人大常委会开展执法检查、专题询问、听取专项工作报告和人大代表视察调研活动，依法接受市人大及其常委会监督。积极支持市政协履行政治协商、民主监督、参政议政职能，认真办理政协提案，积极采纳政协调研成果，重视政协委员反映的社情民意。全年共办理市人大代表建议168件、市政协提案303件，办结率均为100%。广泛听取并吸纳各民主党派、工商联、各人民团体、无党派人士及社会各界人士意见。加快行政审批制度改革，全面推行容缺预审、并联会审、超时默许、缺席默许。以“创先争优深化年”活动为载体，在市直部门开展“改进工作作风、提高行政效能”活动，出台提高行政机关办事效率六条意见，建立项目首席责任人工作制度，推行“一线工作法”，政府执行力有效提高，涌现出市工商局等一批服务基层、服务企业、服务群众的先进集体。严格落实领导干部廉洁自律各项规定，着力推进惩防体系建设，廉政建设和反腐败工作不断加强。

各位代表，随着2011年工作任务的完成，本届政府任期的各项目标已经顺利实现，为在更高起点上推进宁德科学发展、跨越发展奠定了更加坚实的基础。

五年来，经济持续快速发展，质量效益显著提高，实现了“五个倍增”。全市生产总值和人均生产总值增长1倍，财政总收入增长2.1倍，地方级财政收入增长2.2倍，工业总产值跨越千亿台阶、增长2.4倍。三次产业结构不断优化，比重从21.1∶37∶41.9调整为16.8∶48.4∶34.8。

五年来，改革开放持续深化，项目建设卓有成效，实现了“四个突破”。投融资体制改革取得突破，组建市国投公司、城投公司和土地收储中心。外贸外资取得突破，累计外贸出口总额45亿美元，验资口径实际利用外商直接投资2.8亿美元、增长2.5倍。项目建设取得突破，累计完成全社会固定资产投资1543亿元，完成本届政府任期目标的140.3%。品牌建设取得突破，中国驰名商标由零增加到11件；地理标志证明商标23件，居全国各设区市首位。

五年来，基础设施日益完善，城乡面貌焕然一新，实现了“三个提升”。集疏运能力持续提升，新增港口吞吐能力500万吨、铁路里程146公里，改扩建国省干线464公里，建制村通村公路硬化任务全面完成。能源保障能力持续提升，新增电力装机280万千瓦，新建、扩建110千伏及以上变电站13座。城镇化水平持续提升，中心城市累计实施城建项目201个，建成区面积扩大11平方公里，城镇化率提高8.2个百分点。

五年来，人民生活不断改善，社会事业全面进步，实现了“三个同步”。民生投入同步增长，全市各级财政累计用于民生支出达185亿元。居民收入同步提高，职工最低工资标准和公务员工资水平得到提升，城镇居民人均可支配收入、农民人均纯收入分别年均增长12.7%和13.4%。各项社会建设同步推进，文化、教育、卫生、科技事业加快发展，生态环境质量继续保持全省前列。

各位代表，过去的五年，是宁德综合实力提升最快、城乡面貌变化最大、人民群众受益最多的五年。这是中央和省委、省政府正确决策、关心支持的结果，是全市人民在市委坚强领导下团结一致、艰苦奋斗的结果，是市人大、市政协加强监督、鼎力支持的结果。在此，我代表市人民政府，向为环三建设和宁德发展付出辛勤劳动的全市人民，向给予政府工作有力支持和有效监督的人大代表、政协委员、各民主党派、工商联、各人民团体、无党派人士、离退休老同志和社会各界人士，向为宁德发展作出积极贡献的中央及省属驻宁机构、驻宁部队、武警官兵、公安民警，向关心支持宁德发展的港澳同胞、台湾同胞、海外侨胞、国际友人，表示诚挚的感谢和崇高的敬意！

在总结成绩的同时，我们也清醒认识到，在发展中还存在不少困难和问题，主要是：产业基础仍较脆弱，经济总量偏小，财政实力偏弱；资源、环境等制约因素仍然较多，项目落地难问题还比较突出；社会建设相对滞后，民生事业欠账较多；干部作风建设还需进一步加强，不作为和消极腐败现象在个别单位和领域仍有存在，等等。对这些问题，我们一定要认真对待，切实解决。

二、新一届政府工作目标

今后五年，是宁德全力推进环三大开放大开发，加快科学发展、跨越发展的关键时期。政府工作的总体要求是：高举中国特色社会主义伟大旗帜，以邓小平理论和“三个代表”重要思想为指导，深入贯彻落实科学发展观，按照省第九次党代会和市第三次党代会的部署，全力实施环三发展战略，坚持“三化”并举，着力“三群”联动，加快推进经济、政治、文化、社会和生态文明建设，争作全省科学发展、跨越发展的典范，努力发展成为福建新的增长极，与全省同步实现全面建成小康社会目标，为建设更加优美更加和谐更加幸福的宁德而努力奋斗。

今后五年的奋斗目标是：

——综合经济实力显著增强。在确保全面完成“十二五”规划目标任务的基础上，自加压力，努力保持经济发展速度居于全省前列，力争“十二五”期间实现全市地区生产总值四年翻一番，工业总产值和财政总收入五年翻两番，在福建发展格局中位次进一步前移。

——人民生活水平显著提高。城乡居民收入普遍较快增长，社会保障体系和基本公共服务体系更加完善，社会就业更加充分，人居环境明显改善，人民群众安居乐业，生活质量和水平大幅提高，幸福感普遍提升。

——城乡区域发展显著加快。中心城市建成区面积、城市人口实现五年翻一番，基本形成布局合理、功能互补、特色鲜明、绿色宜居的环三城镇体系，城镇化水平力争提高到60%。县域经济规模不断壮大，社会主义新农村建设加快推进，城乡一体化发展取得实质性进展。环三核心区、主体区和统筹区协调发展水平显著提升，形成互动联动发展的新格局。

——生态环境质量显著提升。环境保护工作持续加强，环境污染得到有效治理，主要污染物排放总量和单位地区生产总值能耗控制在省定基数内，基本形成节约资源和保护环境的产业结构、增长方式、消费模式，森林覆盖率继续保持全省前列，生态市建设取得明显成效，人民群众对生态环境的满意度持续提高。

——社会建设管理显著加强。民主法制更加健全，社会管理机制更趋完善，和谐宁德、平安宁德建设再上新水平。义务教育实现基本均衡，城乡公共卫生和医疗服务体系更加完善，文化发展的主要指标在全省居中上水平。区域自主创新能力有效提升，科技进步对经济增长的贡献率达到50%以上，力争进入全国科技先进市行列。

三、2012年政府工作主要任务

今年是新一届政府任期的第一年，也是实施“十二五”规划承上启下的重要一年。做好今年工作，要坚持以科学发展为主题，以转变发展方式为主线，正确理解和把握“稳中求进”的总基调，把“稳中求进”和“好中求快”有机统一起来，围绕经济增长“千百万”目标，以开展“五大战役”拓展年、“三维”项目落实年、园区建设攻坚年、交通港口建设提升年、文化改革发展年等“五个年”活动为抓手，在新的更高起点上全力推进宁德科学发展、跨越发展。

今年经济社会发展的主要预期目标是：地区生产总值增长15%以上，其中第一、第二、第三产业增加值分别增长4.5%、21.5%和11%；财政总收入增长15%以上；全社会固定资产投资增长25%；社会消费品零售总额增长16%，居民消费价格总水平涨幅控制在4%左右；出口总值增长15%，验资口径实际利用外商直接投资增长25%；城镇居民人均可支配收入、农民人均纯收入均增长15%；城镇登记失业率控制在4%以内；人口自然增长率控制在7.6‰以内；单位生产总值能耗和化学需氧量、氨氮、二氧化硫、氮氧化物年度排放量控制在省定目标内。

实现上述目标，重点要抓好八个方面工作：

（一）着力夯实“三农”基础，加快农业现代化步伐

发展壮大农业产业。坚持用工业化理念发展农业，用现代科学技术提升农业，用现代经营方式拓展农业，全面落实强农惠农政策，促进农业增效、农民增收。高度重视粮食安全，稳定粮播面积和粮食产量。围绕构建“两带一区”现代农业产业布局，大力发展精细农业、设施农业和高效生态农业，加快九大特色产业示范基地建设，拓展农产品精深加工，新增产值亿元以上龙头企业3－5家，培育一批农产品知名品牌，扶持农民专业合作社发展，提高农业标准化、规模化、组织化、产业化水平。加快农业综合开发，完成土地整理3.2万亩，新增节水灌溉面积1.4万亩，开发耕地面积1.5万亩。完善农业科技服务体系，推广农业“五新”100项。加强农作物病虫害防控，推进动物疫病防控、农产品质量安全、森林资源保护和农业防灾减灾体系建设。

继续开展扶贫攻坚。切实做好新阶段扶贫开发工作，突出抓好整村推进扶贫工程，加大对海岛偏远地区和库区移民扶持力度，支持革命老区和少数民族地区加快发展。积极推进产业扶贫，加大财政资金和信贷资金投入，扶持发展一批带动贫困户脱贫增收成效明显的项目，增强贫困地区自我发展能力。深入开展文化科技卫生“三下乡”活动，大力实施贫困劳动力转移就业培训“阳光工程”和“雨露计划”，努力提高农民素质。实施新一轮“造福工程”，完成搬迁2万人以上，其中沿海渔民6112人。

大力推进水利建设。加快建设一批国家级小型农田水利重点县，开工建设一批水利重点工程，深化上白石水利枢纽工程前期工作。深入实施农村饮水安全工程，解决30万农村人口饮水安全问题。继续推进水利防洪减灾工程，加快宁德中心城区防洪排涝工程和赛江、霍童溪流域防洪工程建设，继续推进一批农田水利建设工程和海堤、水库除险加固工程。全面实施水土保持综合治理，加强水源保护和利用。

（二）扎实推进转型升级，奋力打造现代产业集群

加快产业结构调整。推进传统产业新型化，以自主创新、技术改造、品牌带动、整合上市为抓手，加快推动电机电器、船舶修造、汽摩配件、食品加工等传统产业转型升级，做大做强传统产业集群。推进临港重化工业高端化，争取开工建设福建鞍钢（宁德）钢铁基地、中海油海西宁德工业区1000万方储油项目，努力打造东南沿海钢铁基地和不锈钢产业基地、海西新兴石化基地。推进新兴产业规模化，建成宁德核电站一期工程，大力开发氧化锆等新材料，培育一批新材料企业集团；着力提升现有医药企业，支持柘荣海西药城、古田药业基地加快建设。发挥海洋资源优势，加快发展海洋新兴产业和涉海现代服务业，形成以沿海重要港湾为依托的海洋经济密集区。

促进产业集聚提升。完善园区产业规划和配套建设，整合提升现有产业园区，着力打造产业集聚发展平台。重点加快溪南、漳湾、赛江、沙埕等四大工业片区建设，山区县集中要素资源配置，重点发展一个工业集中区。坚持“抓龙头、铸链条、建集群”，围绕龙头企业延伸上、中、下游产业链，引导关联企业向园区集聚，培育壮大一批百亿产业集群。大力实施品牌带动战略，再创一批中国驰名商标、福建著名商标和名牌产品。

着力发展现代服务业。全面提升旅游业，大力宣传和培育“宁德世界地质公园”和“闽东北亲水游”旅游品牌，推进太姥山申报世界自然遗产，白水洋、太姥山创建5A级景区，白云山、鲤鱼溪·九龙漈、上金贝、西浦创建4A级景区，加快三都澳、翠屏湖、杨家溪等景区开发建设。大力发展商贸物流业，依托港区、园区、城区和交通道口，规划建设一批现代物流园区和物流中心。有序发展房地产业，认真落实国家稳定房地产市场的各项措施，提高保障性住房供给比例，促进房地产市场平稳健康发展。积极拓展消费领域，发展健身、教育、家政、文化娱乐等服务型消费业态，培育新的消费热点。

（三）发展壮大县域经济，统筹城乡协调发展

提升县域经济实力。实施“大城关”、“大县城”战略，因地制宜、分类指导，打造特色化、品牌化的优势产业，壮大县域经济综合实力，培育各具特色的县域经济体，力争福安市进入全省十强县行列。加强县域交通、电力、水利、信息等基础设施配套，为县域发展提供支撑。继续简政放权，进一步扩大县级管理权限。

做大做强中心城市。高起点、高水平组织实施新一轮宁德城市总体规划和环三城镇群规划。完善老城区功能配套，加快新城区建设步伐，同步推进老城改造和新区建设。加快发展城市公共交通，继续推行公交优先，新建一批公共停车场。继续抓好一批城市路网、市政设施、专业市场、园林绿化、饮水工程、防洪体系和城市综合体建设，提升中心城市功能。强化城市综合管理，继续依法开展“清违”专项行动，提升城市管理和建设水平。

加快推进城镇建设。积极落实“三旧”改造、“退二进三”政策，盘活乡村土地，解决小城镇建设资金问题。推进乡镇机构和小城镇管理体制改革，赋予部分小城镇县级经济社会管理和审批权限，支持有条件的乡（镇）设立一级金库，提高自主发展能力。加快一批重点镇污水、垃圾处理设施建设，启动一批建制镇供水工程建设，深入推进农村环境综合整治，继续开展“家园清洁”行动，新建户用沼气池1000口，改善城乡居民生活环境。加快推进省、市小城镇综合改革试点建设，以试点镇为中心推进周边乡镇联片发展，加快培育一批经济重镇、工贸强镇、旅游旺镇和文化名镇。加强镇村规划编制和管理，三年实现全市村庄规划全覆盖。

（四）强化基础设施建设，切实完善保障支撑体系

完善交通网络。全面推进港口、铁路和公路建设，加快构筑“北承南联、西进东出、通港达海”的现代化立体交通运输体系。大力推进港口开发，加快建设漳湾作业区8#、9#泊位等一批港口码头和深水航道工程，建成白马作业区14#泊位，深化溪南作业区30万吨油品码头前期工作。着力完善陆上交通网络，确保6月份宁武高速公路提前半年建成通车，加快建设京台高速公路宁德段、沈海复线宁连段，开工建设沈海复线贯岭至漳湾段、福寿高速公路，力争开工建设沈海复线闽浙界至太姥山段、溪南疏港高速公路、屏南至古田联络线。深化宁古高速公路前期工作，开展环三疏港高速公路支线网络规划编制。继续抓好一批国省道和县乡村道路改造、建设，加快实施“村村通客车”工程，实现建制村通客车率达92%以上。加快建设合福铁路宁德段、白马港铁路支线，深化衢宁铁路、宁古铁路、溪南铁路支线前期工作，力争早日开工建设。同时，继续推进霞浦军用机场民用扩建工程。

强化能源保障。加快宁德核电站建设，深化海上风力发电宁德示范工程、周宁抽水蓄能项目前期工作。开展新一轮农村电网改造升级，加强中心城区电网建设，开工建设110千伏及以上线路360公里，新建、扩建各级变电站29座。加快一批在建LNG项目建设，深化中心城市到周边县域的LNG管道前期工作。

（五）深化改革扩大开放，激发发展动力活力

继续深化体制改革。深化农村改革，促进农村土地承包经营权规范有序流转，继续推进集体林权制度改革。继续理顺市、县（区）两级行政管理体制，稳步推进综合执法管理体制改革，分类推进事业单位改革，开展新一轮行政审批制度改革。扎实推进投融资体制改革，重点整合盘活国有资产存量，建立以交通、旅游、能源、港口、城建等集团为重点的多元化国有资产投融资平台。认真落实国家、省关于促进中小微企业和非公有制经济发展的政策措施，优化民营经济发展环境，支持民营企业做大做强。实施上市带动战略，加大政策扶持力度，力争我市企业上市有新突破。完善区域自主创新体系，培养和引进一批高层次人才，加强企业、政府、科研院校三方互动，加快推进中科院宁德海西高新技术产业示范园建设，扶持和培育一批高新技术企业。

不断提高招商实效。牢固树立“环境是对外开放第一竞争力”理念，改善优化投资环境。坚持常年招商，积极组织和参加各类境内外招商活动，突出港澳台、长三角、珠三角、浙南及闽南等地招商推介。突出项目质量，加快推进“三维”对接，大力实施“回归工程”，着力引进一批大项目、好项目。抓好签约项目跟踪落实，继续实行项目首席责任人制度和“五个一”工作机制，提高项目履约率，确保“三维”对接合同项目开工率达80%以上。

积极扩大对外贸易。调整优化出口产品结构，提高产品技术含量和附加值。继续推进省电机出口基地建设，深度拓展自贸区等国际市场。继续实施鼓励外贸发展的政策，完善口岸“大通关”工作机制，加强与各类海关特殊监管区的合作，密切税贸、关贸、银贸协作，加强外贸运行监测预警，保持外贸出口持续稳定增长。

促进宁台交流合作。按照“五个共同”原则，拓展宁台产业对接，扩大与台湾在电机、冶金、石化、船舶、旅游和现代农业等方面的交流合作，加快台湾水产品集散中心、台湾渔民创业园规划建设，支持霞浦建设全市性台商投资集中区。继续办好海峡两岸电机电器博览会，落实好“海峡论坛”宁德活动项目，推进陈靖姑、圆瑛、甘国宝等民间信俗和历史名人文化的双向交流。深化与基隆的友好合作，巩固和扩大宁台乡镇对接交流合作成果。

（六）加强社会管理，切实保障和改善民生

健全社会保障体系。积极做好就业工作，多渠道开发就业岗位。适时提高公务员工资水平，不断改善基层一线干部工作条件和生活待遇。继续扩大各类社会保险覆盖范围，扎实开展机关事业单位养老保险制度改革，稳步推进城乡居民社会养老保险工作。加快市县两级救助站建设，加强城乡困难家庭救助和帮扶工作，完善农村五保供养制度。全力推进“金保工程”建设，加快社会保障卡发放。继续推进保障性安居工程建设，开工建设各类保障房8500套。落实“菜篮子”市长负责制，大力推进“万村千乡市场工程”、“家电下乡”工程，进一步完善城乡市场体系。加强市场价格调控监管，加快平价商店、副食品基地建设，落实物价上涨挂钩联动机制，确保物价涨幅控制在全省平均水平以下。

优先发展教育事业。加强校园安全防范和校车安全管理，加快推进中小学校舍安全工程、寄宿制学校建设和城区中小学扩容，实施农村义务教育学生营养改善计划。实施学前教育发展三年行动计划，大力发展普惠性幼儿园，学前三年幼儿入园率达到91.7%。推进义务教育均衡发展，促进城区教育资源整合，完成126所义务教育标准化学校建设，全面实现“双高普九”目标。推进普通高中达标晋级，高中阶段毛入学率达到85%。积极发展职业教育和高等教育，适应产业发展科学设置专业，加快建设宁德师范学院、宁德职业技术学院新校区。扶持发展老年教育、特殊教育和社区教育，着力构建终身教育体系。

提高医疗卫生水平。深化医药卫生体制改革，进一步推进公立医院改革试点工作。实施“全民低成本健康海云工程”，构建区域医疗信息共享平台。加大全科医生和乡村医生培训力度，推进临床医学专业本科定向生委培，实现社区每万人有3名全科医生、每个卫生院有4名全科医生。完成宁德市医院新院的住院楼主体结构、市精神卫生中心病房楼主体工程和市中心血站一期工程建设，新增病床位800张。坚持稳定低生育水平，强化利益导向，综合治理性别比，提高出生人口素质，促进家庭和谐幸福。

加强和创新社会管理。深入开展“六五”普法工作，提高全民法律素质。加强基层社会管理服务，逐步推进社会管理“网格化”。完善“大调解”体系，严格按照“路线图”依法处理信访事项，健全重大项目社会稳定风险评估机制，全面推广和谐征迁工作法。进一步深化“平安宁德”建设，完善社会治安防控体系和公共安全体系，提高应对突发事件能力。严格落实安全生产责任制，加强各领域各行业安全监督管理，防范和遏制重特大事故发生。

（七）大力发展文化事业，加强核心价值体系建设

建设社会主义核心价值体系。坚定社会主义理想信念，大力弘扬以爱国主义为核心的民族精神、以改革创新为核心的时代精神，积极践行社会主义荣辱观。大力弘扬福建精神和闽东精神，进一步筑牢宁德人民团结奋斗的思想基础。深化群众性精神文明创建活动，加强公民道德建设和未成年人思想道德教育，提高公民文明素质和社会文明程度。

着力发展文化事业。深化文化体制改革，加快推进“三网融合”，稳步推进国有文艺团体改革，完善文化市场综合执法运行机制。加强文化基础设施建设，完成市图书馆主体工程建设，继续建设一批乡镇综合文化站，实现所有建制村农家书屋全覆盖。加强非物质文化遗产保护，积极传承闽东红土地文化和畲族文化，引导发展健康向上的网络文化，繁荣发展文学艺术、广播影视、新闻出版事业。大力发展体育事业，重视体育设施建设，广泛开展全民健身活动，努力提高竞技体育水平。

加快发展文化产业。提升文化产业发展规划，加大政策扶持力度，发展壮大传统文化产业，加快发展新兴文化产业，积极引进一批文化产业项目，培育一批骨干文化企业和文化产业园区，推动文化产业跨越发展。重点规划建设一条旅游精品线路、一条工艺美术街、一座茶文化城、一个文化创意产业园、一个文化主题公园、一个影视基地等“六个一”工程，发展一批具有闽东特色的文化产业品牌。

（八）强化环境保障，增强可持续发展能力

推进节能减排。严格环境准入制度，严把新上项目资源能耗关和环境保护关，坚决淘汰落后产能，推广节能技术和清洁生产，大力发展低碳产业和循环经济。加强排污监控和环境监测，强化石材、化工、合成革等重点行业和工业园区污染防治，确保达标排放。加快推进重点节能工程和污染减排项目建设，完善城乡污水垃圾处理及配套设施建设。

加强环境治理。扎实推进生态宁德建设，支持柘荣等县（市、区）创建国家级生态县（市、区），推进环境优美乡镇和生态示范村建设，大力实施“四绿”工程，完成160个绿色村庄建设。加快建立生态补偿机制，抓好饮用水源和重要水源涵养区保护，巩固“两江三溪”流域整治成果，保障生活饮水水质安全。扎实推进殡葬改革，大力治理“青山挂白”。继续加大海上养殖综合整治力度，科学规划重点流域网箱养殖，积极推广生态养殖技术，有效改善水域生态环境。

合理开发资源。严格落实生态控制和耕地保护目标，大力清理已批未建土地，强力推进非法围填海整治工作，提高土地、岸线、滩涂、水域等资源利用率。加快围海造地工程建设，积极推进土地开发整理，确保耕地占补平衡。加强地质灾害防治，推进矿产资源开发整合，规范勘探开发行为。

在抓好八项重点工作的同时，高度重视其他各项事业发展。继续做好国防动员、人民防空工作，加强全民国防教育和民兵预备役建设，深入开展“双拥”共建活动。巩固平等、团结、互助、和谐的民族关系，促进宗教与社会主义社会相适应。认真做好老干部工作，大力发展社会福利、老龄、慈善和残疾人等事业。继续做好人事、编制、统计、审计、档案、外事、侨务、农机、水文、气象、地震、地方志和关心下一代等工作。

四、建设为民负责、高效廉洁的服务型政府

全面实现今年各项目标，任务光荣而艰巨，使命庄严而崇高。必须切实加强政府自身建设，以服务群众、服务基层、服务发展的实绩检验政府工作成效，努力建设人民满意的服务型政府。

（一）解放思想，激发活力。树立终身学习观念，注重政策法规、经济知识和社会管理知识的学习和应用，不断增强驾驭市场经济、服务项目建设、化解社会矛盾、应对突发事件的能力。继续解放思想，更新发展理念，培养超前思维，勇于先行、敢于担当、善于突破，以思想的大解放引领发展大跨越。

（二）转变职能，优化服务。牢记为民服务宗旨，大力推行“四下基层”工作制度和“一线工作法”，深入基层、深入一线、深入群众提供服务、解决问题。规范部门职责权限，完善协调配合机制，解决职能交叉、权责脱节、政出多门、互相推诿等问题，把更多精力和资源集中到社会管理和公共服务上。大力发展电子政务，深化政务公开，畅通民意表达渠道，保障群众合法权益。

（三）勤政敬业，效率优先。继续深入开展“改进工作作风、提高行政效能”活动，扩大人大常委会专题询问、政协民主监督试点范围。更加注重工作落实，雷厉风行、立说立行、一抓到底，确保各项工作部署落实到位、取得实效。更加注重效能提升，深化行政审批制度改革，简化行政审批环节，全面实行容缺预审、并联会审、集中共审，进一步提高行政机关工作效率。更加注重政风建设，改进文风会风，治理“庸懒浮散”，着力解决不作为、慢作为、乱作为等问题，进一步增强服务发展的能力。

（四）依法行政，从严治政。加强政府公务员队伍教育管理，增强公务员依法行政的意识和能力。自觉接受人大及其常委会的工作监督和法律监督，主动接受政协民主监督，强化政府层级监督和专门监督，高度重视社会公众监督和舆论监督，让权力在阳光下运行。加强法治政府建设，严格规范行政执法行为，认真做好行政复议工作。层层落实党风廉政建设责任制，继续推进惩防体系建设，完善公共资源配置、公共资产交易、公共产品生产领域市场化运行机制，严格控制“三公”经费，促进社会公平正义。

各位代表，新的一年，新的起点，新的征程。让我们更加紧密地团结在以胡锦涛同志为总书记的党中央周围，在省委、省政府和市委的坚强领导下，大力弘扬闽东精神，紧紧依靠全市人民，凝心聚力，开拓进取，为建设更加优美更加和谐更加幸福的宁德而努力奋斗，以优异成绩迎接党的十八大胜利召开！
